# Intrinsically Stretchable Organic-Tribotronic-Transistor for Tactile Sensing

**DOI:** 10.34133/2020/1398903

**Published:** 2020-06-24

**Authors:** Junqing Zhao, Tianzhao Bu, Xiaohan Zhang, Yaokun Pang, Wenjian Li, Zhi Zhang, Guoxu Liu, Zhong Lin Wang, Chi Zhang

**Affiliations:** ^1^CAS Center for Excellence in Nanoscience, Beijing Key Laboratory of Micro-Nano Energy and Sensor, Beijing Institute of Nanoenergy and Nanosystems, Chinese Academy of Sciences, Beijing 100083, China; ^2^School of Nanoscience and Technology, University of Chinese Academy of Sciences, Beijing 100049, China; ^3^School of Material Science and Engineering Georgia Institute of Technology, Atlanta, GA 30332, USA; ^4^Center on Nanoenergy Research, School of Physical Science and Technology, Guangxi University, Nanning 530004, China

## Abstract

Stretchable electronics are of great significance for the development of the next-generation smart interactive systems. Here, we propose an intrinsically stretchable organic tribotronic transistor (SOTT) without a top gate electrode, which is composed of a stretchable substrate, silver nanowire electrodes, semiconductor blends, and a nonpolar elastomer dielectric. The drain-source current of the SOTT can be modulated by external contact electrification with the dielectric layer. Under 0-50% stretching both parallel and perpendicular to the channel directions, the SOTT retains great output performance. After being stretched to 50% for thousands of cycles, the SOTT can survive with excellent stability. Moreover, the SOTT can be conformably attached to the human hand, which can be used for tactile signal perception in human-machine interaction and for controlling smart home devices and robots. This work has realized a stretchable tribotronic transistor as the tactile sensor for smart interaction, which has extended the application of tribotronics in the human-machine interface, wearable electronics, and robotics.

## 1. Introduction

Stretchable electronics are grabbing more and more attention for a wide range of applications in wearable devices, soft mechanics, robotic skin, human-machine interfaces, and so on [1–6]. To date, a series of stretchable functional devices have been developed with prominent tactile-sensing properties based on various physical transduction mechanisms such as piezoresistivity [7, 8], capacitance [9], magnetism [10, 11], and optics [12]. However, most of the tactile-sensing mechanisms for stretchable electronics are passive, lacking direct interaction with human/environment [13–17]. This complicates the process of information acquisition and further influences the tactile perception ability of stretchable functional devices. Therefore, developing stretchable electronics with an active sensing mechanism is highly desired.

Since 2012, the triboelectric nanogenerator (TENG) as a new energy technology derived from the Maxwell displacement current has been invented by Wang et al. [18, 19], which can effectively convert mechanical energy into electricity [20–22]. In recent years, tribotronics as a new field has been proposed by using the triboelectric potential generated by TENG to control the carrier transport in semiconductors, which has established the direct interaction mechanism between human/environment and electronics [23–29]. Moreover, a variety of tribotronic functional devices have been demonstrated for tactile perception and control, including smart tactile switch [30], tactile-sensing arrays [31], active modulation of conventional electronics [32], and mechanosensation-active matrix [33]. In addition, tribotronic devices have demonstrated the diversity of material selection [24–36], which is very promising for the intrinsically stretchable electronics for active tactile sensing by further coupling with stretchable materials.

Here, we propose an intrinsically stretchable organic tribotronic transistor (SOTT) without a top gate electrode, which is composed of a stretchable substrate, silver nanowire (Ag NW) electrodes, semiconductor blends, and a nonpolar elastomer dielectric. The drain-source current of the SOTT can be modulated by external contact electrification with the dielectric layer. Under 0-50% stretching both parallel and perpendicular to the channel directions, the SOTT exhibits good output performances. After being stretched to 50% for thousands of cycles, the SOTT can survive with excellent stability. Moreover, the SOTT can be conformably attached to the human hand, which can be used for tactile signal perception in human-machine interaction and for controlling smart home devices and robots. This work has realized a stretchable tribotronic transistor as the tactile sensor for smart interaction, which has extended the application of tribotronics in human-machine interface, wearable electronics, and robotics.

## 2. Results and Discussion

### 2.1. Fabrication of the Stretchable Tribotronic Transistor

Poly(3-hexylthiophene-2,5-diyl) (P3HT), as a polymer semiconductor, has a high hole mobility and a low band gap width [37]. The P3HT nanofibril (P3HT-NF) combined with the stretchable elastomer materials has high stretchability, which is good for developing stretchable semiconductor devices [38, 39]. Among a lot of elastomers, the polydimethylsiloxane (PDMS) with a simple preparation process can sustain large strains. Moreover, the PDMS has a good triboelectric property, which is good for tribotronic devices [40–42]. Ag NWs have good conductivity, which have been widely used in the field of stretchable electrodes [43, 44]. Therefore, in order to obtain highly stretchable SOTT, we exploit the P3HT nanofibril-percolated PDMS rubber composite as a stretchable semiconductor, the Ag NWs dispersed within the PDMS as a stretchable conductor, and the PDMS as a gate dielectric. Through the contact electrification between the external triboelectric layer and the PDMS gate dielectric layer, the drain-source current of the transistor can be modulated.

**Figure 1:**
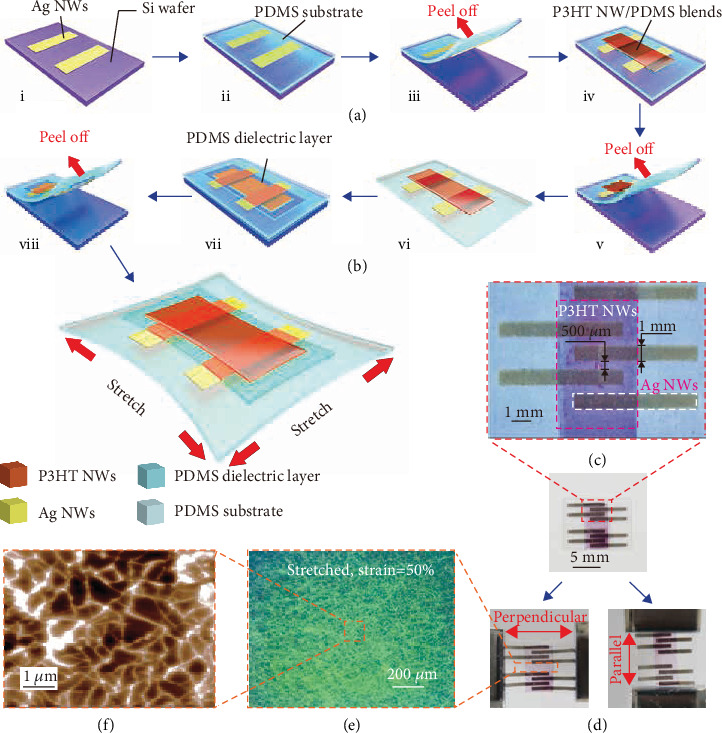
Overview of the SOTT. (a) Fabrication process of the SOTT without a top gate electrode. (b) Schematic structure of the SOTT. (c) Optical images of the SOTT. (d) The stretched SOTT both parallel and perpendicular to the channel directions. (e) Optical microscope images of P3HT NF/PDMS film at 50% strain, with (f) an AFM phase image showing the morphology of P3HT NF/PDMS film.

The schematic illustration of the fabrication process for the SOTT is shown in Figure 1(a). The detailed process is elaborated in Materials and Methods. The SOTT consists of a stretchable substrate, Ag NW electrodes, semiconductor blends, and a nonpolar elastomer dielectric, which is fabricated throughout a sequential lamination transfer process. To prepare the stretchable drain and source electrodes, Ag NWs were spray coated onto an octadecyltrimethoxysilane- (OTS-) pretreated silica wafer through a shadow mask (i), then followed by embedding into a nonpolar elastomeric PDMS substrate (ii). To build an ohmic contact between the Ag NW electrodes and the semiconductor, the PDMS substrate with Ag NW electrodes was immersed into HAuCl_4_·H_2_O solution for the formation of gold nanoparticles on the Ag NWs by an in situ reduction method (iii). Scanning electron microscopy (SEM) images of the stretchable Ag NW electrodes before and after immersion into HAuCl_4_·H_2_O solution indicate the successful formation of gold nanoparticles, as shown in Figure S1. The stretchable semiconductor blends were prepared by a cooling and heating process. Briefly, P3HT was dissolved in m-xylene at 70°C and then cooled to room temperature for the formation of P3HT nanofibrils. After mixing with m-xylene-diluted PDMS, the P3HT NF solution was subsequently spin-coated onto the drain-source electrodes through a polyimide shadow mask to achieve a patterned semiconductor layer (iv). Contained in the transparent PDMS, the semiconductor layer has revealed great optical transparency, which is important for the application in wearable and bionic electronics (Figure S2). The stretchable dielectric layer, which is composed of PDMS, was spin-coated onto a polytetrafluoroethylene (PTFE) block and then transferred onto the P3HT NF/PDMS semiconductor layer to form the final SOTT (v and vii). The complete structure of the SOTT shown in Figure 1(b) was obtained by peeling off the whole device from the PTFE block (viii), which has demonstrated a simple structure without a top gate electrode. The channel length is about 500 *μ*m, as shown in Figure 1(c). Since all components of the device are stretchable, the prepared SOTT can be stretched both parallel and perpendicular to the channel directions. Figure 1(d) shows the optical graphs of a stretched device in two directions. As clearly seen from the graphs, the device can be deformed without physical damage upon stretching. Moreover, optical microscopy and atomic force microscopy (AFM) images of the P3HT NF/PDMS blends have demonstrated that the semiconductor blends can be stretched without any obvious cracks under 50% strain, as shown in Figures 1(e) and 1(f), which is very helpful for promoting the stretchability of the SOTT.

### 2.2. Mechanism and Performances of the Stretchable Tribotronic Transistor

**Figure 2:**
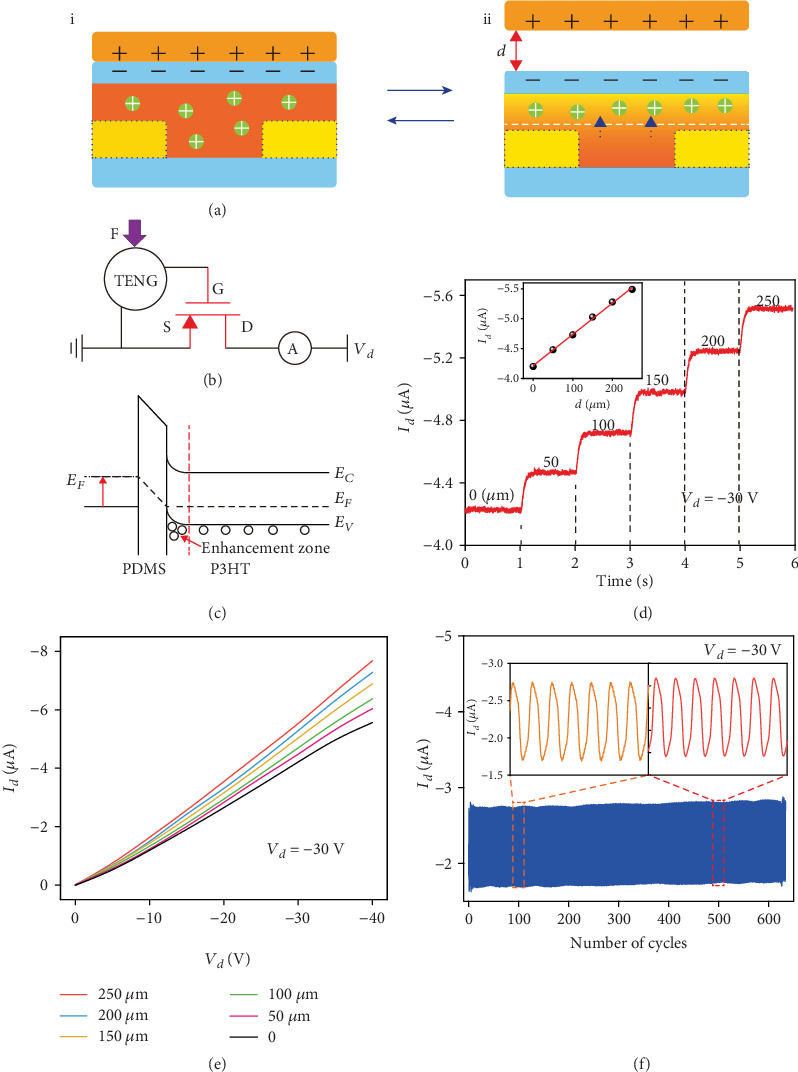
Working mechanism and output characteristics of the SOTT. (a) Working principle of the SOTT. (b) Equivalent circuit and (c) energy band diagrams of the SOTT. (d) *I*_*d*_ changes of the SOTT at different separation distances, the drain-source voltage (*V*_*d*_) remains -30 V. The inset is the *I*_*d*_‐*d* transfer characteristics. (e) *I*_*d*_‐*V*_*d*_ output characteristics with different separation distances. (f) Durability test of the SOTT.

The working mechanism of the SOTT is presented in Figure 2(a). The drain and source electrodes of the SOTT are connected with a voltage source. An aluminum (Al) film, as an external triboelectric layer, fully contacts with the dielectric layer in the initial state for electrification as shown in Figure 2(a), i. The Al film is electrified with positive charges while the PDMS dielectric layer with negative charges for the difference in charge affinities. Owing to the electrostatic balance, electrical potential difference is not applied to the channel region, and no obvious changes take place in the drain current. When the Al film gradually separates from the PDMS dielectric layer by an external force as shown in Figure 2(a), ii, negative charges on the dielectric layer surface will induce an inner charge polarization, which will build an inner electric field across the channel and the dielectric surface, leading the holes to accumulate at the interface of the channel and the dielectric layer. As a result, an enhancement zone is achieved in the p-type P3HT NF/PDMS channel, and the drain current is increased. The enhancement zone and the current will be enhanced until a maximum separation distance of the Al film is reached. When the Al film starts to come back to the original station, the inner electric field will be decreased for the depressed inner charge polarization, resulting in the previous accumulated holes diffusing away from the interface of the channel and the dielectric layer. Therefore, the enhancement zone is depressed, while the hole concentration in the interface and the drain current are decreased. Once the Al film reaches its initial state, the hole concentration in the interface and the drain current recover to the original value. It is worth noting that a depletion zone will be formed in the channel and the current will be decreased when a film with strong electronegativity contacts and then separates from the PDMS dielectric layer, such as the fluorinated ethylene propylene (FEP) film. This is the basic operation mechanism of the SOTT, which can be equivalent to a circuit in Figure 2(b). The electrostatic potential generated from the contact electrification between the Al film and the dielectric layer is equivalent to an external gate voltage, which is illustrated in the energy diagram shown in Figure 2(c). Figure S3 shows the simulation results of the electrostatic potential generated from the contact electrification between the Al film and the dielectric layer, which indicates that the triboelectric potential is dependent on the separation distance of the Al film. As shown in Figure S4, the drain current changes with the reciprocating motion of the Al film, showing a consistency with the working mechanism that we discussed above. In addition, we have increased the velocity of the contact-separation movement, until the time of rise and fall for the drain current signal is not changed. The corresponding waveform of *I*_*d*_ is shown in Figure S4, which indicates that the response and recovery times are 80 ms and 90 ms, respectively, which exhibits that the device has a small hysteresis and shows a potential of SOTT to construct sensing electronics.

To better evaluate the performance of the SOTT, the electrical characteristics of the SOTT at a different separation distance of the Al film without any mechanical stretching are systematically studied. The separation distance of the Al film is precisely controlled by a linear motor.  Figure 2(d) and Figure S5 show the relationship between the drain current and the separation distance. The inset image is the corresponding transfer characteristics of the SOTT, and the drain current changes with the traditional gate voltage are shown in Figure S6. The drain current is obtained at a drain voltage of -30 V. With the separation distance increases from 0 to 250 *μ*m, the drain current is increased from -4.15 *μ*A to -5.55 *μ*A.  Figure 2(e) contains the output characteristic curves of the SOTT at a different separation distance from 0 to 250 *μ*m. The drain current increases with the increasing separation distance, with the drain voltage swept from 0 to -40 V, which is in good accordance with the working mechanism analyzed above. Moreover, as shown in Figure 2(f), the current can be modulated by the periodic contact-separation motion of the external triboelectric layer for more than 600 cycles with little hysteresis, showing a high stability of the device.

**Figure 3:**
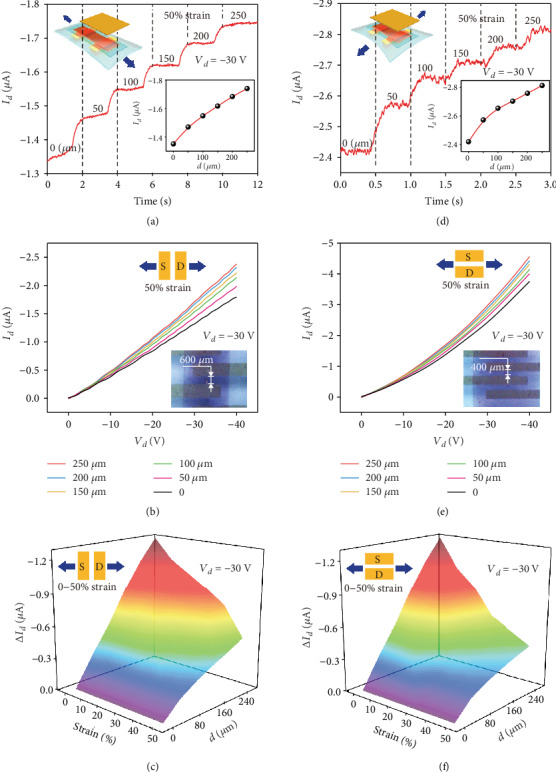
Output characteristics of the stretched SOTT. (a) *I*_*d*_ changes with separation distance and (b) output characteristic curve of the SOTT after 50% mechanical strain was imposed parallel to the channel direction. The inset in (a) is the *I*_*d*_‐*d* transfer characteristics, and the insert in (b) is the optical graph of the stretched SOTT. (c) Δ*I*_*d*_ changes with separation distance at different levels of mechanical strain in parallel to channel direction. (d) *I*_*d*_ changes with separation distance and (e) output characteristic curve of the SOTT after 50% mechanical strain was imposed perpendicular to the channel direction. The insets in (d) and (e) are the *I*_*d*_‐*d* transfer characteristics and optical graphs of the stretched SOTT, respectively. (f) Δ*I*_*d*_ changes with separation distance at different levels of mechanical strain in perpendicular to the channel direction.

To examine the performances of the SOTT under mechanical strain, the electrical characteristics of the SOTT with increasing separation distance are collected by stretching the devices both parallel and perpendicular to the channel directions. Note that the SOTT was fabricated with stretchable components; the stretching strain distributed in the device is effectively suppressed and assumed to be distributed across the whole device, as shown in Figure S7.  Figure 3(a) shows the transfer characteristics of the SOTT under 50% stretching strain parallel to the channel direction. More results are illustrated in Figure S8, which has shown that the SOTT can survive after being stretched parallel to the channel direction. When the SOTT is stretched up to 50%, an increase in the drain current from -1.35 *μ*A to -1.75 *μ*A is obtained at the separation distance from 0 to 250 *μ*m. Besides, the output characteristic curve of the SOTT at 50% strain in the parallel stretching direction is shown in Figure 3(b). More results are depicted in Figure S9. With the increases of the separation distance, the drain current rises within a drain voltage of 0 to -40 V for the stretched SOTT. The insert image in Figure 3(b) is the optical microscope images of stretched SOTT parallel to the channel direction. It can be clearly observed that the channel was stretched to about 600 *μ*m without any rupture accrued. On the basis of these transfer curves, the variations of the drain current at different separation distance and stretching strain were calculated, as shown in Figure 3(c). The device can maintain good performance when the device was stretched by up to 50% parallel to the channel direction.

Compared to the parallel direction stretching, an increase from -2.42 *μ*A to -2.85 *μ*A in the drain current was obtained at the separation distance from 0 to 250 *μ*m when the SOTT was stretched to 50% perpendicular to the channel direction, as shown in Figure 3(d). The device also has good output characteristics when the drain voltage swept from 0 to 40 V after being stretched, as shown in Figure 3(e). The transfer and output characteristics of the SOTT at different stretching strains from 10% to 40% perpendicular to the channel direction are shown in Figure S10 and S11, respectively. Also, the variations of the drain current at different separation distances and stretching strains perpendicular to the channel direction were calculated, as depicted in Figure 3(f). An ideal modulation performance of the SOTT can be also maintained up to 50% stretching strain. Moreover, the distance resolution of the SOTT is illustrated in Figure S12, showing that the SOTT has excellent distance resolution in the initial state and the stretched state. All these results suggest that the intrinsically stretchable organic tribotronic transistor can maintain good output performances after being stretched, which may promise a bright future of tribotronics in stretchable smart sensing electronics.

**Figure 4:**
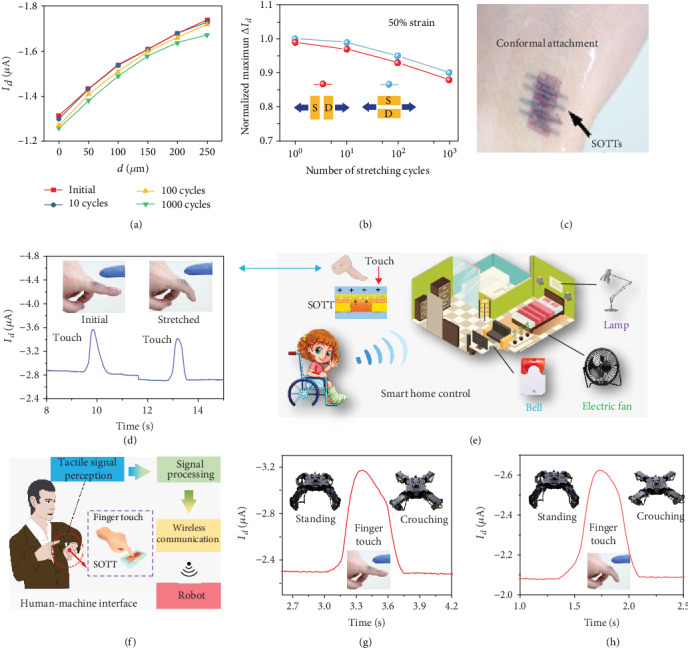
SOTT for tactile perception in smart home equipment and robot controls. (a) *I*_*d*_‐*d* fitted curves of the SOTT that was cycled to stretched state. (b) Normalized maximum current during cycling at 50% strain in parallel and perpendicular to the channel directions. (c) A Photo of the SOTT attached conformably on the human hand. (d) Photographs and corresponding signals of the initial and stretched SOTT attached conformably on the finger. (e) Schematic diagram of the SOTT as the tactile sensor in smart home control for both healthy and disabled people. (f) Schematic diagram of the SOTT as the tactile sensor in human-machine interaction for robot control. Photograph and corresponding signals of the (g) initial and (h) stretched SOTT attached conformably on the finger as tactile sensor for robot control.

Specifically, the SOTT shows unprecedented robustness when stretched repeatedly both parallel and perpendicular to the channel directions. As exhibited in Figure 4(a), only a small shift can be observed in the transfer characteristics curves of a SOTT that was cycled to the stretched state. The normalized maximum current variations during cycling at 50% stretching strain parallel and perpendicular to the channel directions are exhibited in Figure 4(b). After one thousand stretching cycles, the current variation at the maximum separation distance only decreased by about less than 10% both parallel and perpendicular to the channel directions, showing a high stretchability of the SOTT. Moreover, as shown in Figure 4(c), the SOTT can be conformably attached to the human hand due to its stretchability, which is very beneficial for skin-inspired devices.

Owing to the simple form of the structure, the merit of stretchability, and the retention of performance, the SOTT is very promising for active tactile sensing. Hence, in order to fulfill the potential of SOTT in active tactile-sensing applications, the SOTT was used for controlling smart home devices. As shown in Figure 4(d), the SOTT is integrated on a finger as a tactile sensor. The finger can be divided into two states. In the initial state, the finger is so straight that the SOTT is not stretched. Positive charges will be induced on the dielectric layer of the SOTT, and a current increase can be observed when another finger touches the SOTT. When the finger is bent and the SOTT is stretched, a touch can still be perceived by the stretched SOTT, which has shown a high potential of the SOTT as a tactile sensor for a smart control system regardless of pristine or stretched states. Through the tactile perception of the SOTT, the common home devices, such as a table lamp, a bell, and an electric fan, can be wirelessly controlled by a finger touch, as shown in Figure 4(e), which presents many potential applications for the SOTT in daily life, such as the self-care for the disabled. Moreover, as shown in Figure 4(f), tactile sensing can promote the function of human-machine interaction, such as wirelessly controlling a robot. A SOTT is directly attached to the finger for robot control. A current change signal can be observed when the finger touches the SOTT, which is treated as a tactile-sensing signal. The signal is the original blinking signal from the SOTT, followed by the signal after being filtered, amplified, and relay-converted. Then, the output terminal of the latching relay is connected with a microcontroller which can send the robot control instructions through a wireless transmitting module. The robot posture will be controlled when a signal is received by the wireless receiving module on the robot. As a response to the tactile, the robot changes the posture from standing to crouching, as shown in Figure 4(g). When the SOTT is in the stretched state, the posture of the robot can still be controlled by a finger touch, as depicted in Figure 4(h), which has shown great application prospects of the SOTT in smart interaction. All these results have demonstrated the remarkable application potential of the SOTT in human-machine interface, wearable electronics, intelligent skin, and robotics.

## 3. Conclusions

In summary, we have proposed an intrinsically stretchable organic tribotronic transistor without a top gate electrode, which consists of a stretchable substrate, silver nanowire electrodes, semiconductor blends, and a nonpolar elastomer dielectric. Using the contact electrification between the Al film and the PDMS dielectric layer, the drain-source current of the SOTT is increased (-4.15 *μ*A to -5.55 *μ*A) as the separation distance of the Al film goes up (0 to 250 *μ*m), with an excellent stability for more than 600 cycles. Composed of stretchable materials, the SOTT can be stretched both parallel and perpendicular to the channel directions, with excellent output performances at the strain range from 0 to 50% along two directions. The SOTT can be stretched for thousands of cycles with less than 10% decrease in output performances, showing an excellent robustness of the SOTT. Moreover, the SOTT can be conformably attached to the human hand. Through the tactile perception of the SOTT, the common smart home devices and the robot have been successfully controlled. This work has realized a stretchable tribotronic transistor as the tactile sensor for smart interaction, which has extended the application of tribotronics in human-machine interface, wearable electronics, and robotics.

## 4. Materials and Methods

### 4.1. Materials

Anhydrous m-xylene (>99%), acetone (>99.9%), gold chloride trihydrate (HAuCl_4_·H_2_O, >99.9%), octadecyltrimethoxysilane (OTS), and anhydrous ammonia (NH_4_OH, 28%) were all from Sigma-Aldrich and used as received. Regioregular P3HT (rr-P3HT) was from Xi'an p-oled company. Ag NW (~99.5%) solution (average diameter and length are 120 nm and 20 mm, respectively) was from Jiangsu XFNANO Materials Company and used as received.

### 4.2. P3HT NW/PDMS Blend Semiconductor Solution

Regioregular P3HT was dissolved in m-xylene at 70°C and then cooled to room temperature. PDMS (Dow Corning SYLGARD 184, crosslinker : prepolymer = 1 : 10 (*w*/*w*)) was also dissolved in m-xylene. The two solutions were blended together and then aged at room temperature for 1 h to promote crystalline P3HT formation in the blend solution. Before spin-coating, the solution was put in a refrigerator (-15°C) for 30 min and then placed on a table to increase its temperature to 25°C.

### 4.3. Fabrication of the Stretchable Electrodes

First, the Ag NW solution was spray coated using a commercial airbrush through a shadow mask onto a Si wafer that has been pretreated with octadecyltrimethoxysilane and dried for 10 min at 60°C. Then, a PDMS solution [10 : 1 (*w*/*w*) prepolymer/curing agent] was spin-coated on the patterned Ag NW electrodes at 300 rpm for 60 s and followed by curing for 4 hours at 60°C to solidify. The solidified PDMS was peeled off from the Si wafer, and the patterned Ag NW electrodes were embedded into the PDMS. To build an ohmic contact between the electrodes and the semiconductor, the PDMS with embedded Ag NW electrodes was immersed into 0.5 mM HAuCl_4_·H_2_O solution for 2 min and then followed by dipping into NH_4_OH solution (28%) for 1 min. The stretchable electrodes were completed by rinsing in water and drying.

### 4.4. Fabrication of the SOTT

The P3HT-NF/PDMS blend solution was spin-coated on the Ag NW electrodes at 2000 rpm for 60 s and dried for 30 min at 90°C. Then, a PDMS (crosslinker : prepolymer = 1 : 10 (*w*/*w*)) solution was spin-coated on a PTFE cube and dried at 60°C overnight as a dielectric. The PDMS with embedded electrodes and patterned semiconductor were laminated on the PDMS dielectric. Finally, the PDMS dielectric, the semiconductor layer, and the PDMS dielectric were peeled off together to complete the SOTT fabrication.

### 4.5. Material and Device Characterizations

The surface morphology of the P3HT-NF/PDMS composite was characterized using an optical microscope (Zeiss, Axioscope AI) and an AFM (Veeco Dimension 3000) under the tapping mode. The microstructures of the stretchable electrodes were characterized by a SEM (XL-30SFEG, Philips). The electric output characteristics of the devices were measured by using a Stanford SR570. Cyclic mechanical stretching and releasing tests were performed by using a linear motor.

## Supplementary Materials

Supplementary materials Figure S1: SEM images of the Ag NWs/PDMS before and after HAuCl_4_ treatment. Figure S2: UV-vis transmittance spectra of the P3HT NF/PDMS blends. Figure S3: the distribution and theoretical calculation results of the triboelectric potentials simulated by COMSOL Multiphysics software. Figure S4: *I*_*d*_ changes of the SOTT during periodical contact-separation processes with the demonstrations of the response (left) and recovery time (right). Figure S5: transfer curves of the transistor with traditional gate voltage. Figure S6: *I*_*d*_ changes of the SOTT at different separation distances; the drain-source voltage (*V*_*d*_) remains -30 V. The inset is the *I*_*d*_‐*d* transfer characteristics. Figure S7: simulation of mechanical behavior with different semiconductor layer. Figure S8: characteristics of the SOTT under different mechanical strain. Figure S9: *I*_*d*_ output characteristics of the SOTT under different strain. Figure S10: characteristics of the SOTT under different mechanical strain. Figure S11: *I*_*d*_ output characteristics of the SOTT under different strain. Figure S12: distance resolution of the SOTT in the (a) initial state and stretched to 50% in (b) parallel and (c) perpendicular to the channel directions.
