# Supplementary material for: Intrinsically Stretchable Organic-Tribotronic-Transistor for Tactile Sensing
Source: Research (Wash D C). 2020 Jun 24;2020:1398903. doi: 10.34133/2020/1398903 (PMC7333181; doi:10.34133/2020/1398903)
Supplement: Supplementary materials — Figure S1: SEM images of the Ag NWs/PDMS before and after HAuCl4 treatment. Figure S2: UV-vis transmittance spectra of the P3HT NF/PDMS blends. Figure S3: the distribution and theoretical calculation results of the triboelectric potentials simulated by COMSOL Multiphysics software. Figure S4: Id changes of the SOTT during periodical contact-separation processes with the demonstrations of the response (left) and recovery time (right). Figure S5: transfer curves of the transistor with traditional gate voltage. Figure S6: Id changes of the SOTT at different separation distances; the drain-source voltage (Vd) remains -30 V. The inset is the Id‐d transfer characteristics. Figure S7: simulation of mechanical behavior with different semiconductor layer. Figure S8: characteristics of the SOTT under different mechanical strain. Figure S9: Id output characteristics of the SOTT under different strain. Figure S10: characteristics of the SOTT under different mechanical strain. Figure S11: Id output characteristics of the SOTT under different strain. Figure S12: distance resolution of the SOTT in the (a) initial state and stretched to 50% in (b) parallel and (c) perpendicular to the channel directions. [file 1398903.f1.docx]

**Supplementary Materials**

**Intrinsically Stretchable Organic-Tribotronic-Transistor for Tactile Sensing**

Junqing Zhao^1,2, §^, Tianzhao Bu^1,2, §^, Xiaohan Zhang^1, 2^, Yaokun Pang^1, 2^, Wenjian Li^1, 2^, Zhi Zhang^1, 2^, Guoxu Liu^1, 2^, Zhong Lin Wang^1, 2, 3^, Chi Zhang^1, 2, 4, *^

^1^ CAS Center for Excellence in Nanoscience, Beijing Key Laboratory of Micro-nano Energy and Sensor, Beijing Institute of Nanoenergy and Nanosystems, Chinese Academy of Sciences, Beijing 100083, P. R. China

^2^ School of Nanoscience and Technology, University of Chinese Academy of Sciences, Beijing 100049, P. R. China

^3^ School of Material Science and Engineering Georgia Institute of Technology, Atlanta, GA 30332, USA

^4^ Center on Nanoenergy Research, School of Physical Science and Technology, Guangxi University, Nanning 530004, China

Correspondence should be addressed to Chi Zhang; czhang@binn.cas.cn.

^§^These authors contribute equally to this work.


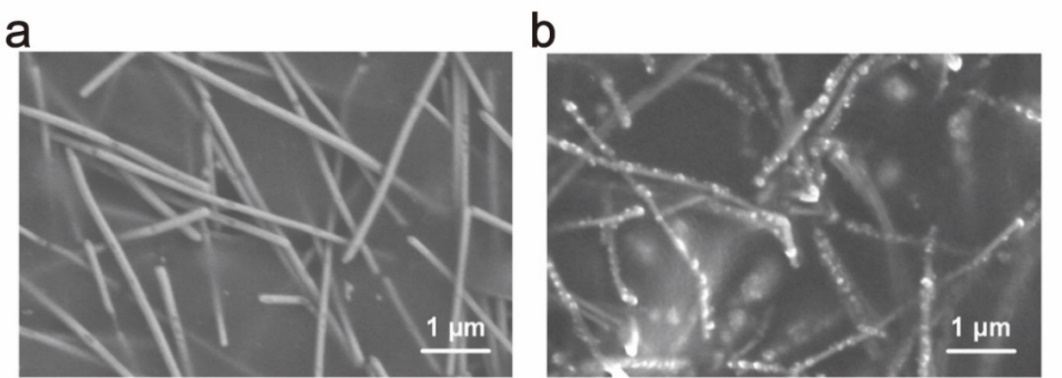


**Figure S1.** **SEM images of** **the Ag NWs/PDMS before and after HAuCl_4_ treatment.** (a) SEM image of the Ag NWs embedded in PDMS. (b) SEM image of the Ag NWs/PDMS after HAuCl_4_ treatment.


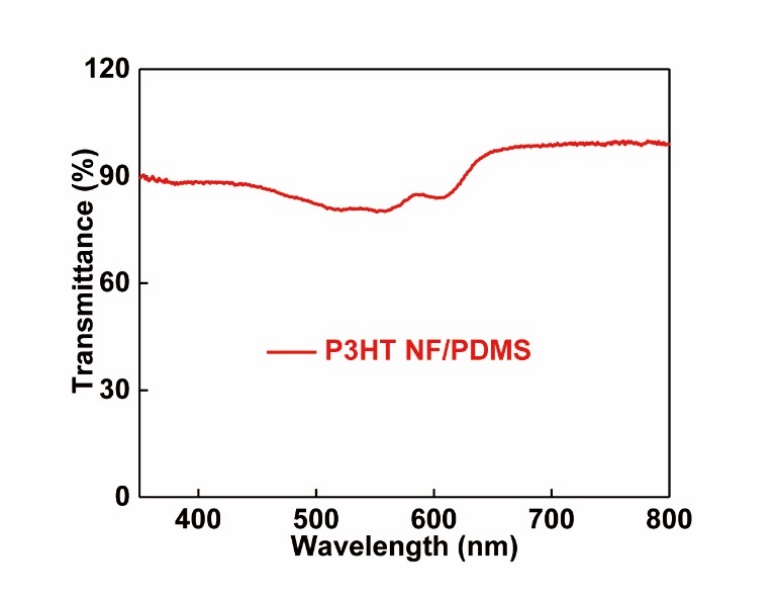


**Figure S2**. UV-vis transmittance spectra of the P3HT NF/PDMS blends.


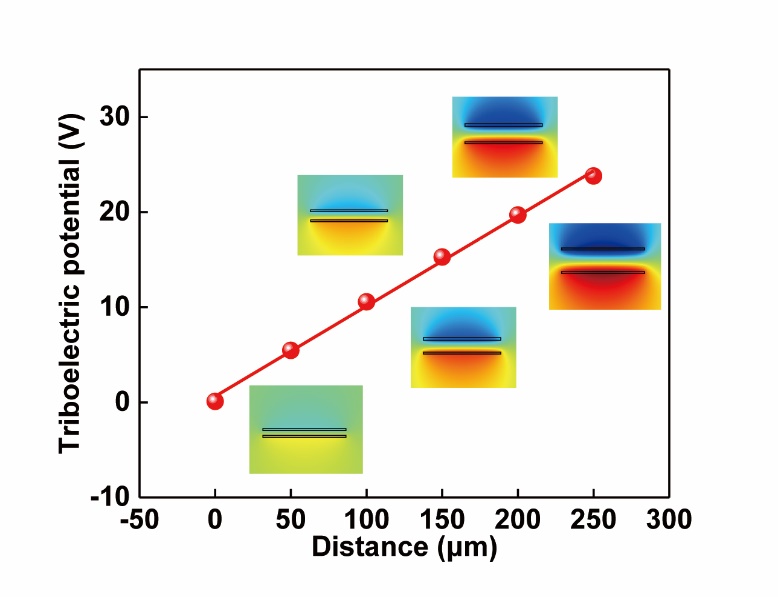


**Figure S3**. The distribution and theoretical calculation results of the triboelectric potentials simulated by COMSOL multiphysics software.


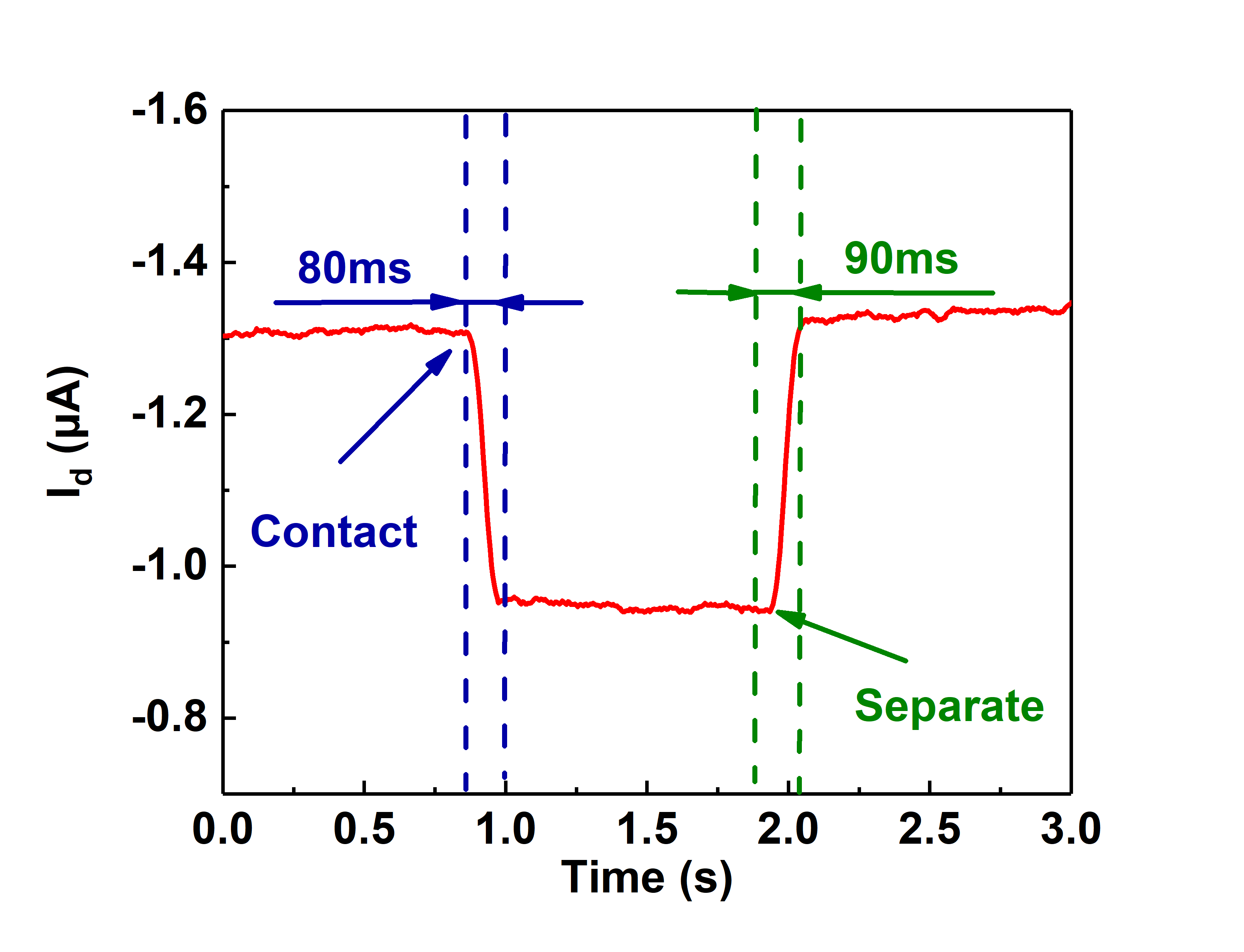


**Figure S4.** I_d_ changes of the SOTT during periodical contact-separation processes with the demonstrations of the response (left) and recovery time (right), respectively.

**
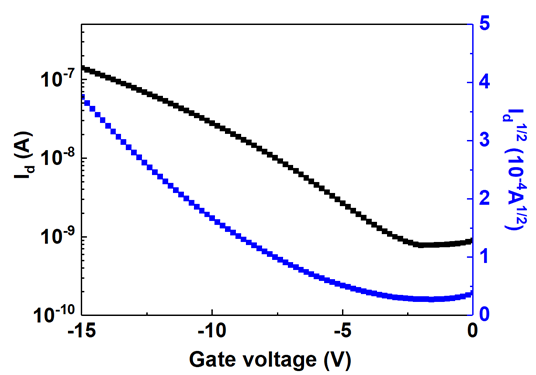
**

**Figure S5** Transfer curves of the transistor with traditional gate voltage.


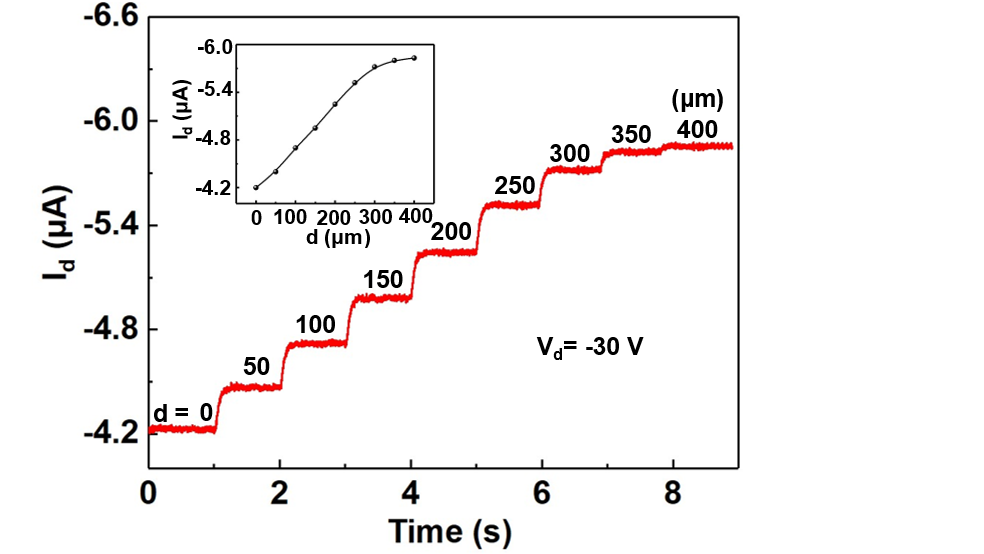


**Figure S6** I_d_ changes of the SOTT at different separation distances, the drain-source voltage (V_d_) remains -30 V. The inset is the I_d_-d transfer characteristics.

**
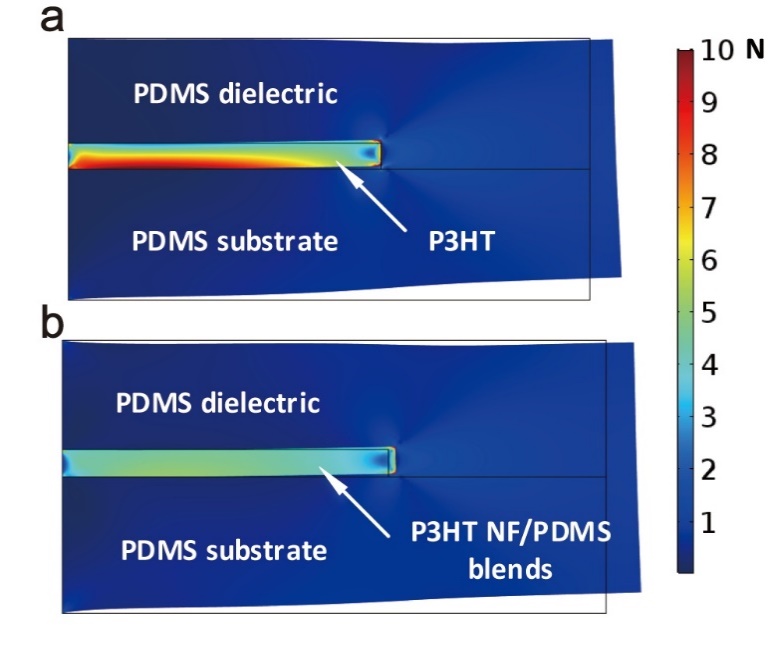
**

**Figure S7. Simulation of mechanical behavior with different semiconductor layer.** (a) P3HT, (b) P3HT NF/PDMS blends.


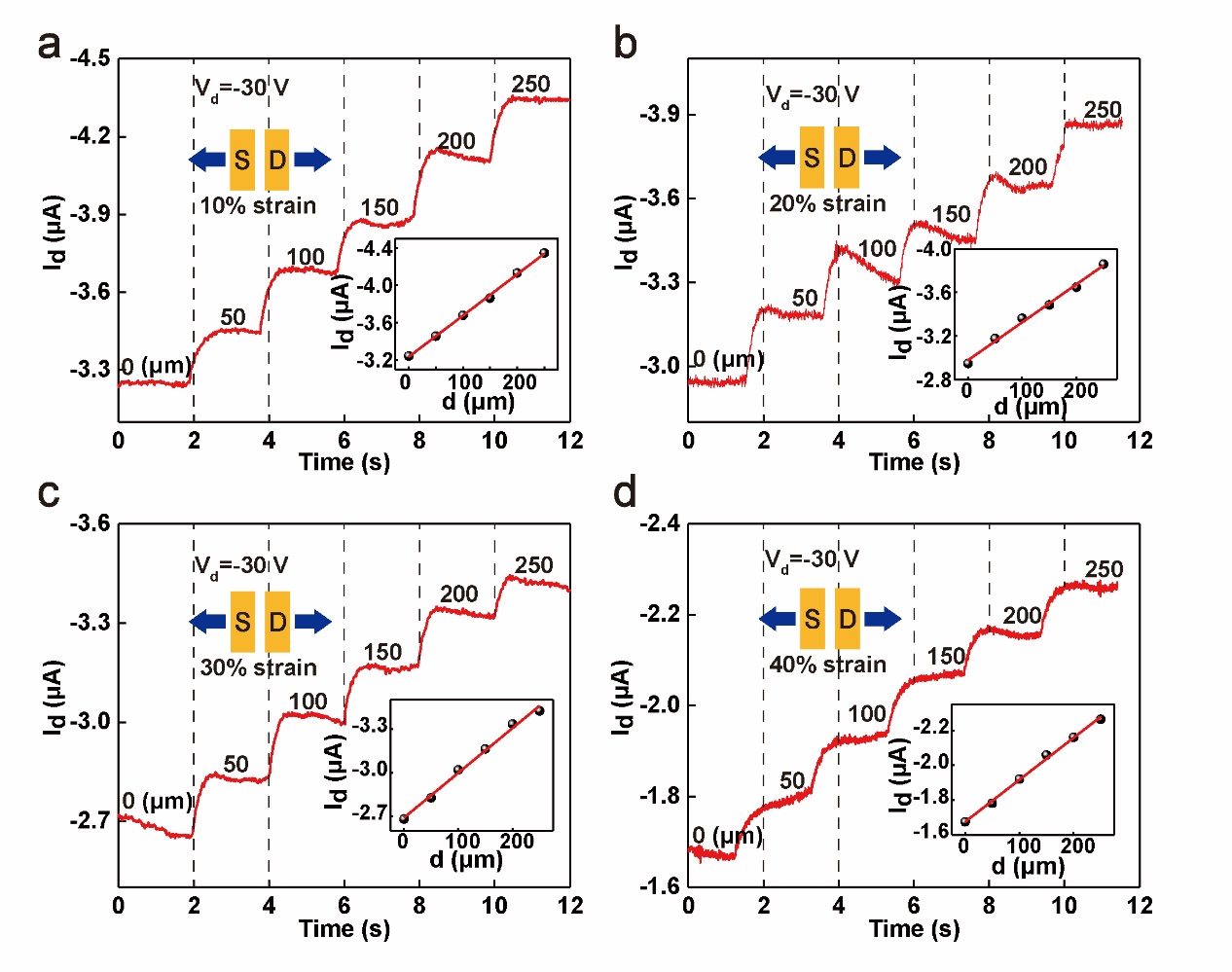


**Figure S8. Characteristics of the SOTT under different mechanical strain.** Corresponding I_d_ changes with separation distance after (a) 10%, (b) 20%, (c) 30%, (d) 40% mechanical strain was imposed parallel to the channel direction. The insets are the I_d_-d fitted curves.


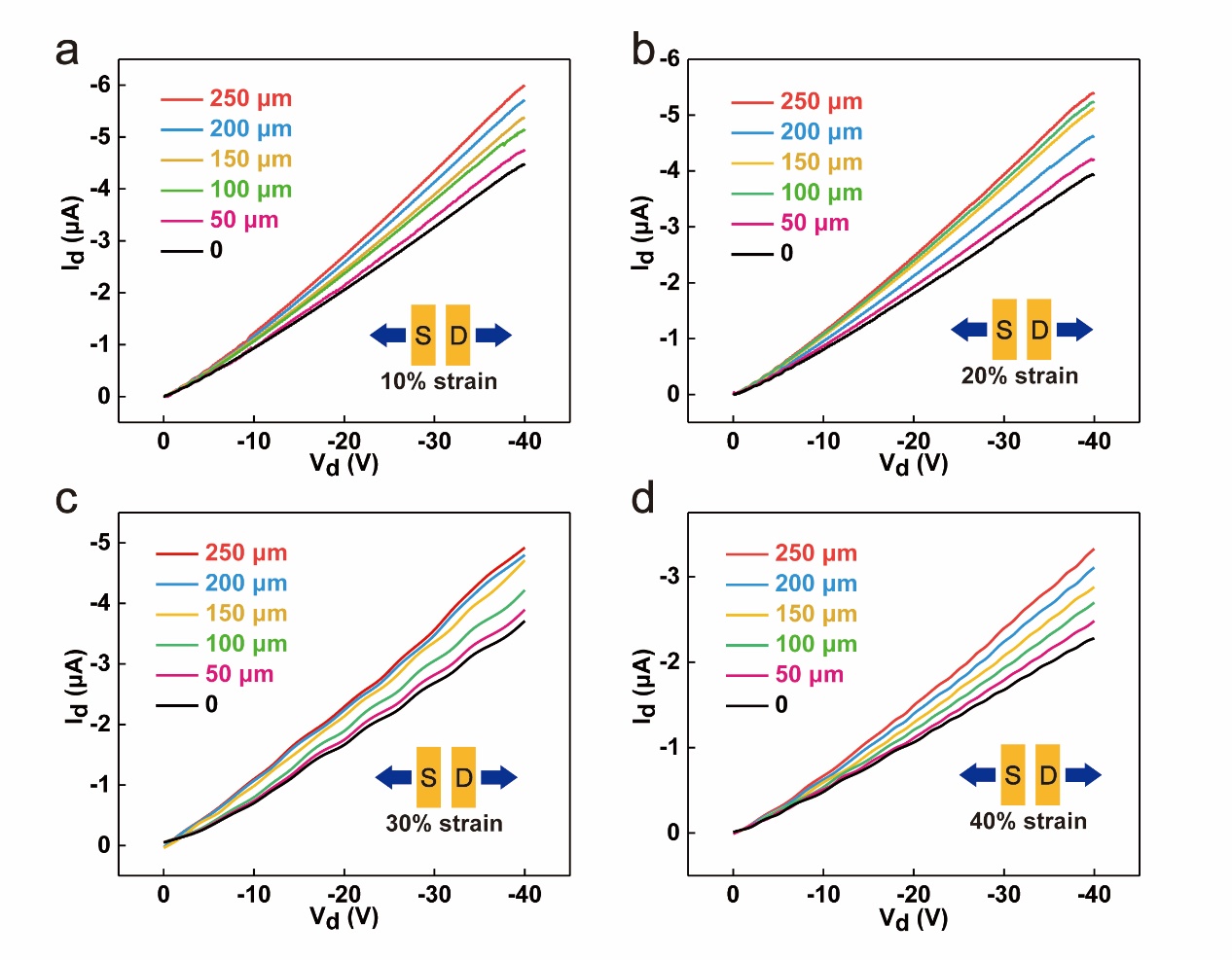


**Figure S9. I_d_ output characteristics of the SOTT under different strain**. I_d_-V_d_ output characteristics of the SOTT with separation distance under (a) 10%, (b) 20%, (c) 30%, (d) 40% strain in parallel to the channel direction.


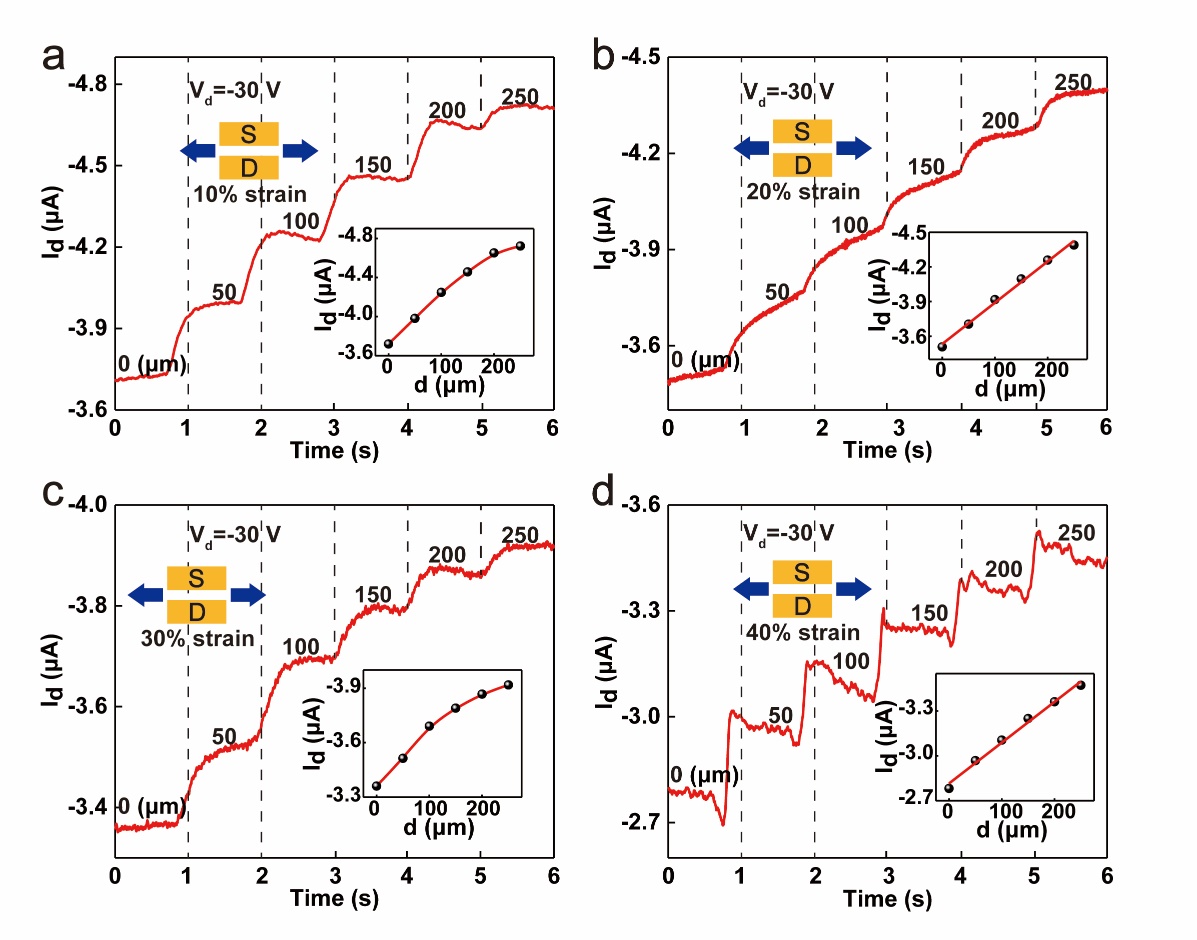


**Figure S10. Characteristics of the SOTT under different mechanical strain**. Corresponding I_d_ changes with separation distance after (a) 10%, (b) 20%, (c) 30%, (d) 40% mechanical strain was imposed perpendicular to the channel direction. The insets are the I_d_-d fitted curves.


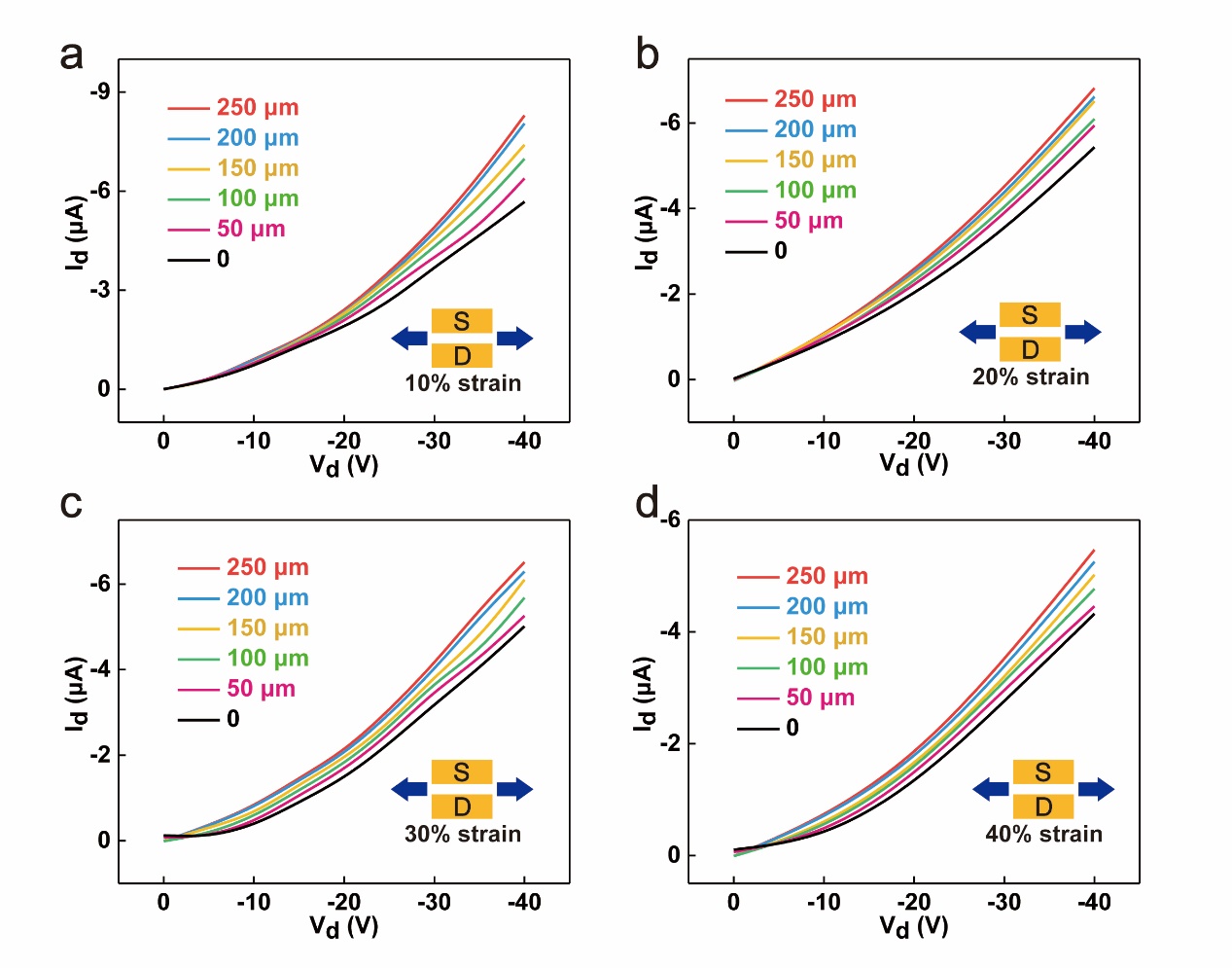


**Figure S11. I_d_ output characteristics of the SOTT under different strain.** I_d_-V_d_ output characteristics of the SOTT with separation distance under (a) 10%, (b) 20%, (c) 30%, (d) 40% strain in perpendicular to the channel direction.


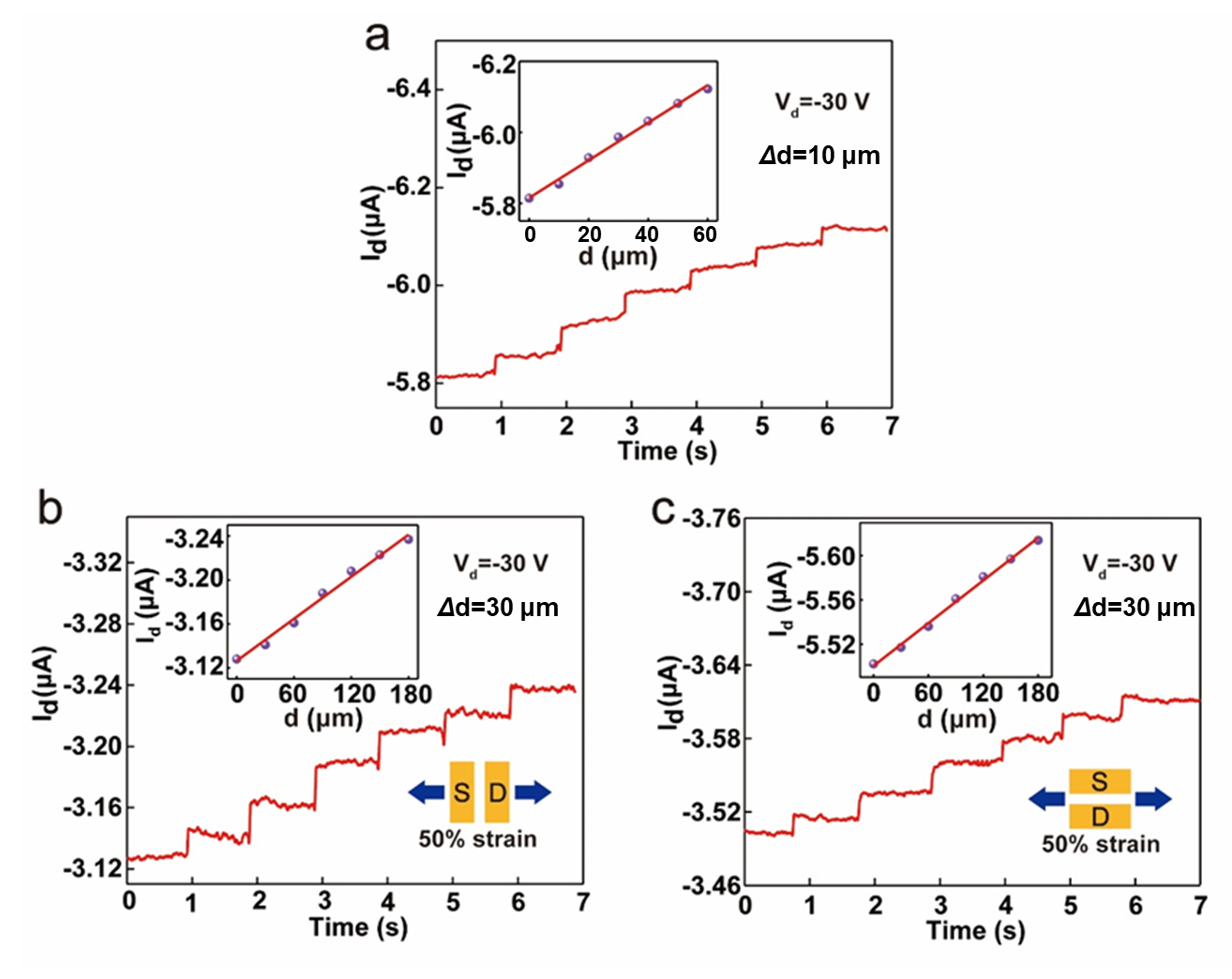


**Figure S12**. Distance resolution of the SOTT in the (a) initial state and stretched to 50% in (b) parallel and (c) perpendicular to the channel directions.
